# On autoethnography

**DOI:** 10.1177/1466138117723354

**Published:** 2017-09-06

**Authors:** Annelies Moors

**Affiliations:** Universiteit van Amsterdam, the Netherlands

When I read this article, I was surprised to see that the authors use the term autoethnography for something that seems to me much closer to a social experiment. They argue that autoethnography ‘uses the researcher’s personal experiences as primary data’ and that ‘the consensus view is that autoethnography relies on using and analyzing the researcher’s own experiences’. I doubt whether there is such a consensus, and if there were, I would like to question it. Anthropologists engage in participant observation as a main method to learn about the lives of others. Many of us live with our interlocutors, listen to them, engage in some of their activities and reflect on our experiences. Would this mean anthropologists always also engage in autoethnography?

My main argument to consider such a broad use of the term autoethnography problematic is that it does not take the positionality of researchers seriously enough. The reflexive turn of the 1980s (as in the seminal *Writing Culture* volume, Clifford and Marcus, 1986) has engendered considerable debate amongst anthropologists. Some have argued that an exaggerated focus on one’s own experiences has come at the expense of paying attention to the lives of others. Others have pointed out that this reflexivity has overlooked both the conditions of possibility of engaging in anthropological fieldwork and the positionality of researchers, that is, the importance of unequal access to material and political resources and to social and cultural capital. This is convincingly concretized in *Women Writing Culture* (Behar and Gordon, 1995), the feminist response to *Writing Culture* that had excluded the work of women anthropologists with the argument that their work was insufficiently innovative.

Using the term autoethnography begs the question how this is different from engaging in participant observation, a way of working in which ethnography is grounded. My point is that how we learn to do anthropological fieldwork depends very much on the pre-existing relation between anthropologists and their interlocutors. When one is a relative outsider to the lifeworld of one’s interlocutors, participant observation involves developing relations of trust, forms of closeness and empathy by participating in their lifeworlds (even if this always remains limited). But if one starts off as relatively close to, or part of, a particular lifeworld, and already has acquired forms of experiential, embodied knowledge, one has a different point of departure. Then one moves from participating to reflecting upon one’s experiences. The latter is what I would consider autoethnography. Lumping together all research that reflects on the researcher’s experiences as autoethnographic fails to recognize how one’s ability or inability to ‘leave the field’ structures one’s experiences. Although there are, of course, grey areas, I agree with Crawley (2012), who argues for limiting the use of the term autoethnography to an analysis of those experiences that did not first occur as part of a fieldwork project.^1^

Let me briefly turn to one of the two cases mentioned in the article, that of wearing a face-veil, and explain why I consider this a social experiment rather than a form of autoethnography. The aim of the researcher is to get as close as possible to what women who wear a face-veil experience in daily life, in particular, the effects of the often hostile responses of the public. Whereas I understand the desire to reveal this hostility and how this affects the self, I am uncomfortable with the lack of discussion about how the researcher’s experiences inevitably differ from those of women who wear a face-veil out of conviction. After all, when a researcher starts to wear a face-veil, she opts for a particular garment that she can take off whenever she feels the urge or the need to do so. But when one does so as an act of worship, as an intrinsic part of one’s telos, then it is quite likely that this different intention structures one’s experiences of public hostility in a different way (e.g. considering it a test) and the pressure to take it off will have different existential consequences.

This then also raises another question. What is the value of engaging in this social experiment over and above doing research with face-veiling women? Does it really provide us with greater insight than if one listens carefully to the narratives of the women concerned? My concern here is that the main advantage may well be the kind of authority one can claim in public debate. In spite of a growing distrust of academic research, the general public still ascribes greater credibility to the words of a white non-Muslim researcher than to those of face-veiling women who are always already considered as biased.

## Note

1 With thanks to Anna Aalten for this reference.
